# Supplementary material for: Reconciling Mining with the Conservation of Cave Biodiversity: A Quantitative Baseline to Help Establish Conservation Priorities
Source: PLoS One. 2016 Dec 20;11(12):e0168348. doi: 10.1371/journal.pone.0168348 (PMC5173368; doi:10.1371/journal.pone.0168348)
Supplement: S1 Dataset — (ZIP) [file pone.0168348.s002.zip › Taxa/Serra Norte/SN_2013/N4WS/Lista N4WS-74.pdf]

CAVIDADE N4WS-0074

Classe	Ordem	Fam/Outros	Gên/Outros	Espécie	S-U	U-S	Zon.
Arachnida	Acari	Anystidae	*Erythracarus*	*nasutus*		X	E
Arachnida	Acari	Argasidae	*Ornithodoros*	sp.1		X	P
Arachnida	Acari	Laelapidae		sp.2		X	P
Arachnida	Amblypygi	Phrynidae	*Heterophrynus*	*longicornis*		1	E
Arachnida	Araneae	Corinnidae		jovem	1		E
Arachnida	Araneae	Ctenidae		jovem	1		E
Arachnida	Araneae	Drymusidae		jovem	X		E
Arachnida	Araneae	Filistatidae		sp.1		X	P
Arachnida	Araneae	Mysmenidae	*Microdipoena*	sp.1		X	E
Arachnida	Araneae	Ochyroceratidae	*Ochyrocera*	sp.1	X	X	E
Arachnida	Araneae	Oonopidae	*Neoxyphinus*	sp.1		X	E
Arachnida	Araneae	Pholcidae	*Leptopholcus*	sp.1	X	X	E
Arachnida	Araneae	Prodidomidae		jovem		X	E
Arachnida	Araneae	Scytodidae	*Scytodes*	jovem	1		E
Arachnida	Araneae	Theridiidae	*Achaearanea*	sp.1	X	X	E
Arachnida	Araneae	Theridiosomatidae	*Plato*	sp.1		X	P
Chilopoda	Scolopendromorpha	Cryptopidae	*Cryptops*	sp.1	1		E
Diplopoda	Polydesmida	Paradoxosomatidae		sp.1	3		E
Entognatha	Collembola	Paronellidae		sp.1	X	X	E
Entognatha	Diplura	Campodeidae		sp.1		X	E
Entognatha	Diplura	Projapygidae		sp.2		X	E
Insecta	Blattodea			jovem	3	1	E
Insecta	Coleoptera	Tenebrionidae		jovem	X		E
Insecta	Diptera	Cecidomyiidae	Cecidomyiinae	sp.	X	X	P
Insecta	Diptera	Ceratopogonidae		sp.	X		E
Insecta	Diptera	Dolichopodidae		sp.	X		E
Insecta	Diptera	Psychodidae	*Micropygomyia*	*pilosa*		X	E
Insecta	Diptera	Psychodidae	*Sciopemyia*	*sordellii*	X	X	E
Insecta	Diptera	Sciaridae	*Bradysia*	sp.	X		E
Insecta	Diptera	Tipulidae	Tipulinae	sp.	X		E
Insecta	Heteroptera	Lygaeidae	Rhyparochrominae	sp.1	X	X	E
Insecta	Heteroptera	Reduviidae	Emesinae	jovem	2		E
Insecta	Homoptera	Cixiidae		sp.1		X	P
Insecta	Homoptera	Cixiidae		sp.3	X		E
Insecta	Hymenoptera	Diapriidae		sp.2		X	P
Insecta	Hymenoptera	Formicidae	*Camponotus*	sp.1	X	X	E/P
Insecta	Hymenoptera	Formicidae	*Gnamptogenys*	*striatula*	X		E
Insecta	Hymenoptera	Formicidae	*Hypoponera*	sp.1		X	E
Insecta	Hymenoptera	Formicidae	*Pachycondyla*	*striata*		1	P
Insecta	Hymenoptera	Formicidae	*Solenopsis*	sp.2		X	E
Insecta	Isoptera	Termitidae	*Nasutitermes*	sp.	X	X	P
Insecta	Lepidoptera			jovem		X	E
Insecta	Neuroptera	Myrmeleontidae		jovem		1	P
Insecta	Orthoptera	Phalangopsidae	*Phalangopsis*	sp.	9	3	E
Insecta	Psocoptera	Psocomorpha		jovem		X	E
Insecta	Psocoptera	Psyllipsocidae		jovem		X	E/P
Malacostraca	Isopoda	Armadillidae		sp.1	X		E
Amphibia	Anura	Strabomantidae	*Pristimantis*	cf. *fenestratus*	14		E
Amphibia	Anura			sp.5		1	E

Mammalia	Chiroptera	Emballonuridae	*Peropteryx*	sp.		2	P
